# Second Thoughts on First Thoughts

**DOI:** 10.1093/asjof/ojaa044

**Published:** 2020-11-07

**Authors:** Lorne K Rosenfield

**Affiliations:** Division of Plastic and Reconstructive Surgery, University of California, San Francisco (UCSF), San Francisco, CA, USA

## Abstract

This article represents the inaugural edition of our new “Second Thoughts on First Thoughts” section with Dr Aly as our featured master surgeon. In this article we will explore the evolution of his now renowned surgical strategies from their birth, through their active tinkering, to their ongoing tweaking, even today. The reader will then come away with a deeper understanding of Dr Aly’s “learning curve” and in so doing be spurred forward on their own learning curves.

How many times have you been surprised to learn that a surgeon had changed their technique just when you were getting the hang of the one they spoke or wrote about? Or worse, how about when you don’t hear about their modifications until long after you’ve already experienced your own technical issues? Well, this new journal section will hopefully bring you some solace, perspective, and inspiration (Video 1). These seemingly annoying edits actually offer us a valuable window into the creative process of change: where the surgeon instinctively elicits second thoughts about their first thoughts. And these second thoughts about their operative technique are, perforce, usually better than their first and reflect their dogged procedural tinkering and resultant incremental advancements forged on their journey to “better.” And if we were to string all of these small practice-induced “upgrades” together, we will plot what I call their “surgical learning curve.” It is precisely this creative process that this article will expose.^1^ We’ll review the surgeon’s first thoughts about the surgical solutions they initially employed for a particular procedure. Then, we will focus on their second thoughts about how those same strategies were gradually tweaked to achieve ever better results. Finally, we close with their “third” thoughts about the unsolved hurdles that they still face and the relentless efforts they are making to pursue “better” still.

Speaking of the relentless pursuit for excellence, our inaugural featured surgeon, Dr Al Aly’s own second thoughts were always inspired by his mentor Dr Millard’s admonition to “know the ideal beautiful normal.” ^2^ After diligently rereading everything Al has ever written and then interviewing him—and reinterviewing him—I can confidently state that he is surely the contemporary torchbearer of this surgical proverb. It prodded Dr Aly to alchemize its essence into his own set of successful body contouring techniques that should equally stand the test of time.

## FIRST THOUGHTS

Dr Aly’s first thoughts elucidate the surgical challenges he observed at the start of his journey as he grappled to improve the outcomes in the field of weight-loss surgery.

### First Thoughts on Body Contouring

Dr Aly noted that the contemporary techniques that he had “inherited” and dutifully applied were delivering unpredictable and incomplete results. Dr Aly describes how his patient selection lacked the necessary discrimination to promote better outcomes and fewer complications, his inconsistent design and markings led to less than predictable results and scar placement, and overall, his surgical approach promoted operative times that were simply too long.

## SECOND THOUGHTS

Dr Aly’s second thoughts about these first thoughts are what prodded him to start modifying the contemporary body contouring techniques, particularly in the brachioplasty and the body lift.

### Seconds Thoughts on the Brachioplasty

1. **Length of the Incision**: Initially, Dr Aly would end the medial incision as a “T” to simply remove the dog-ear, but he found his results lacked proper correction of the redundant skin beyond the arm. Therefore, he began to extend the incision onto the chest to more comprehensively repair the deformity.
2. **Timing of Wound Closure**: He originally used to leave the first arm open and return to close it only after completing the second side. But a genuinely exceptional case permanently changed his approach: when he returned to the first arm to complete its closure, he noted that the patient’s radial pulse was absent. After a few deep, panic-filled, breaths, he elected to reopen the wound, which blessedly restored the pulse. And then a few days later, he returned to the operating room for a secondary closure. From then on, he has always closed the brachioplasty wound *immediately* following excision to avoid any opportunity for the mounting edema.
3. **Ideal Shape of the Brachium**: Al at first was not satisfied with his brachioplasty results, which tended to look natural due to a decidedly tubular shape. So, he decided to conduct a study to help understand and define the “beautiful normal” brachium. It was then that he realized he had been creating *cylindrical* arms rather than the more beautiful, normal shape of a *con*e: wider at the axilla and narrower at the elbow. Thus, began the technical advancement with the removal of more skin at the elbow than the axilla to realize this more pleasing contour. Specifically, he would start distally, carefully pinching and tailor-tacking the arm closed, effectively pushing the excess proximally, to mitigate both the common distal dog-ear and ensure the creation of this more natural cone shape.

### Second Thoughts on the Body Lift

1. **Operative Planning**: He at first attempted to conduct the body lift surgery solo, but after too many gruelingly long days, he realized that he needed a second pair of hands. So, joining forces with Dr Albert Cram at the University of Iowa, Dr Aly effectively cut his operative time in half. And from then on, this dynamic duo went on to literally rewrite the book on body lifts.
2. **Operative Technique and Patient Positioning**: Initially, Dr Aly started his patients in a prone position to otherwise prevent the potential disruption of an abdominal repair conducted first. However, he soon discovered that by doing so, the posterior deformity was being repaired at the expense of what would ultimately be the more visible result: the anterior reconstruction. That is, Al found that the initial prone repair would often advance some of the deformity anteriorly, marring the result aesthetically. Thus, from then on, Dr Aly’s strategy evolved to start the surgery in the supine position to ensure as complete and attractive an anterior repair as possible.^3^
3. **Scar Placement**: At first, Al’s primary objective was to simply remove the excess skin. However, it soon became apparent this goal fell short when he witnessed postoperative scars that would wander aimlessly and ultimately land asymmetrically and/or too high or too low. So, Al went back to the literature and happened upon the concept of the *zones of adherence* as described by Ted Lockwood. At the same time, Al began to appreciate the malleability or lack thereof of the skin, by what he coined the “translation of pull”.^2^ When Al combined this concept with Lockwood’s novel zones of adherence principle to his surgical planning and procedures, it facilitated a more precise divining of the final wound placement and eventual scar location.^4^
4. **Patient Selection**: On account of his witnessing his share of complications,^1^ Al’s patient selection criteria for his extensive body contouring procedures have definitely become stricter over the years: he no longer operates on the obese patient, the diabetic, or the smoker, all candidates he believes promise essentially a 100% incidence of complications. Al points out that the ostensible “courageous” surgeries that he is known to do belie his inherently conservative nature. Having operated on every patient variety and experienced every untoward result, he is now far more discriminating and equally transparent with patients about their operative candidacy and realistic goals, respectively. That said, regardless of how far from the “ideal” the patient may be, Al feels strongly that as long as he can realize a significant difference in their shape, he is willing to operate. So, in that sense, Al is effectively cherry-picking his cases—not postoperatively as is often the case—but more efficaciously...preoperatively!
5. **Panniculectomies:** Al describes that he originally was enticed by the attraction of the panniculectomy’s potential to deliver dramatic results, he now feels very different. He has since found these kinds of procedures, in light of the untreated convexity secondary to the attendant massive intraabdominal contents, to be palliative at best. And Al also admits that he later appreciated another insight into this problem from one of his mentors that there is essentially a 100% recurrence rate, particularly in those pannuses with extensive chronic edema.
6. **Chemo-Prophylaxis:** Al witnessed his share of pulmonary embolisms in his body contouring practice despite following the prevailing venous thromboembolism (VTE) prophylaxis guidelines. So, about mid-career, he changed course with the inception of the epidural as his routine form of surgical anesthesia which also delivered better patient comfort and greater postoperative mobility. Since doing so, he has, to date, not seen another thromboembolism.^5^
7. **Seroma Prevention and Treatment**: Since the beginning of time, Al admits to have endured far too many seromas. But this infernal problem did prompt him to test many preventative tactics over the years. In fact, although at the start he tried every type and number of drains, he now no longer uses them. Instead, he has now become a big proponent of progressive tension sutures applied aggressively—particularly on back wounds where he used to see the highest incidence of seromas—in combination with compression garments. And if he does witness a recalcitrant seroma, he now will often use oral diuretics to more successfully “dry the well.”

## THIRD THOUGHTS

Dr Aly’s third thoughts about his second thoughts highlight the results with which he remains not quite satisfied and the strategies with which he is still tinkering in his ongoing efforts to improve upon his outcomes.

### Third Thoughts on the Brachioplasty

1. **Length of the Incision**: Dr Aly is continuing to extend his incisions onto the chest as far as is necessary to try to realize an ever more complete excision of the prevailing redundancy.
2. **Timing of Wound Closure**: He still abides by the strict mandate that the first brachioplasty be closed immediately after excision.
3. **Ideal Shape of the Brachium**: Dr Aly persists at trying to create the more “perfect normal” brachium by continuing to study his results, as he has done since the beginning of time to further tweak his marking strategies.

### Third Thoughts on the Body Lift

1. **Operative Planning**: Dr Aly continues to hone the “team” approach for greatest efficiency and effectiveness, particularly in most of the arduous cases.
2. **Operative Technique and Patient Positioning**: His surgical sequence has always been supine–lateral–lateral to ensure maximal correction where it is most needed: at the anterior abdomen. However, more recently, he has been trying what he calls a “fast-prone” sequence: “fast” to reduce risks of this position and “prone” to more efficiently treat the patient with a modest anterior abdominal deformity.

And this same “fast-prone” approach has lent itself to the application of a new abdominoplasty technique that Dr Aly he is presently trying called the Transverse plication, limited Undermining, full Liposuction, neoUmbilicoplasty, and low transverse Abdominoplasty (TULUA) method. Al is testing this technique on the lower BMI patient with only a modest diastasis. This novel and one could argue, the counter-intuitive procedure involves a more limited flap dissection to just the infraumbilical zone, and a fascial plication performed *horizontally*. So far, Dr Aly has experienced very gratifying results with fewer complications than experience with his traditional approach.^6^

1. **Scar Placement**: He continues to strive for greater control over the “landing” of the eventual scars by ever more respecting and applying the concepts of the skin’s zones of adherence and translation of pull.
2. **Patient Selection**: Dr Aly still endeavors to be increasingly discriminating about who he is willing to operate upon. Comorbid attributes such as diabetes, smoking, and obesity have increasingly become contraindications to major body contouring surgery.
3. **Panniculectomies:** He remains stalwart in his conviction to no longer perform major panniculectomies on the body contouring patient.
4. **Chemo-Prophylaxis:** Until proven otherwise, Dr Aly sees his epidural anesthesia strategy as his permanent VTE prophylactic strategy.
5. **Seroma Prevention and Treatment**: Again, in light of Dr Aly’s self-effacing confession of his singularly robust and annoyingly persistent experience with seromas, he still soldiers on in his quest for the most efficacious strategies for prevention and treatment. He describes how he most recently has been assaying the idea of an injection or 2 of steroids into the seroma pocket—with dramatically effective resolution so far.

## CONCLUSIONS

“Nobody ever calls their own kids ugly.” On first blush, after beginning this article with such a high brow admonition by Millard, concluding with this maxim by Dr Aly may seem like we have descended from the sublime to the ridiculous. But this trenchant insight actually so perfectly encapsulates Dr Aly’s modest but powerfully practical surgical philosophy. And conveniently, this admirable approach to surgery and more importantly to our results mirrors the very essence and purpose of this new ASJ section: if we as plastic surgeons are to effectively advance our art, we must always, with great honesty, call out our results when they are not as aesthetic or as uncomplicated as we had wished and planned: we must always have second thoughts about our first thoughts.
